# Supplementary material for: Interferon priming is essential for human CD34+ cell-derived plasmacytoid dendritic cell maturation and function
Source: Nat Commun. 2018 Aug 30;9:3525. doi: 10.1038/s41467-018-05816-y (PMC6117296; doi:10.1038/s41467-018-05816-y)
Supplement: Supplementary file 1 — Supplementary Information [file 41467_2018_5816_MOESM1_ESM.pdf]

Supplementary Information

Interferon priming is essential for human CD34+ cell-derived plasmacytoid dendritic cell maturation and function

Laustsen et al.

Supplementary Figure 1

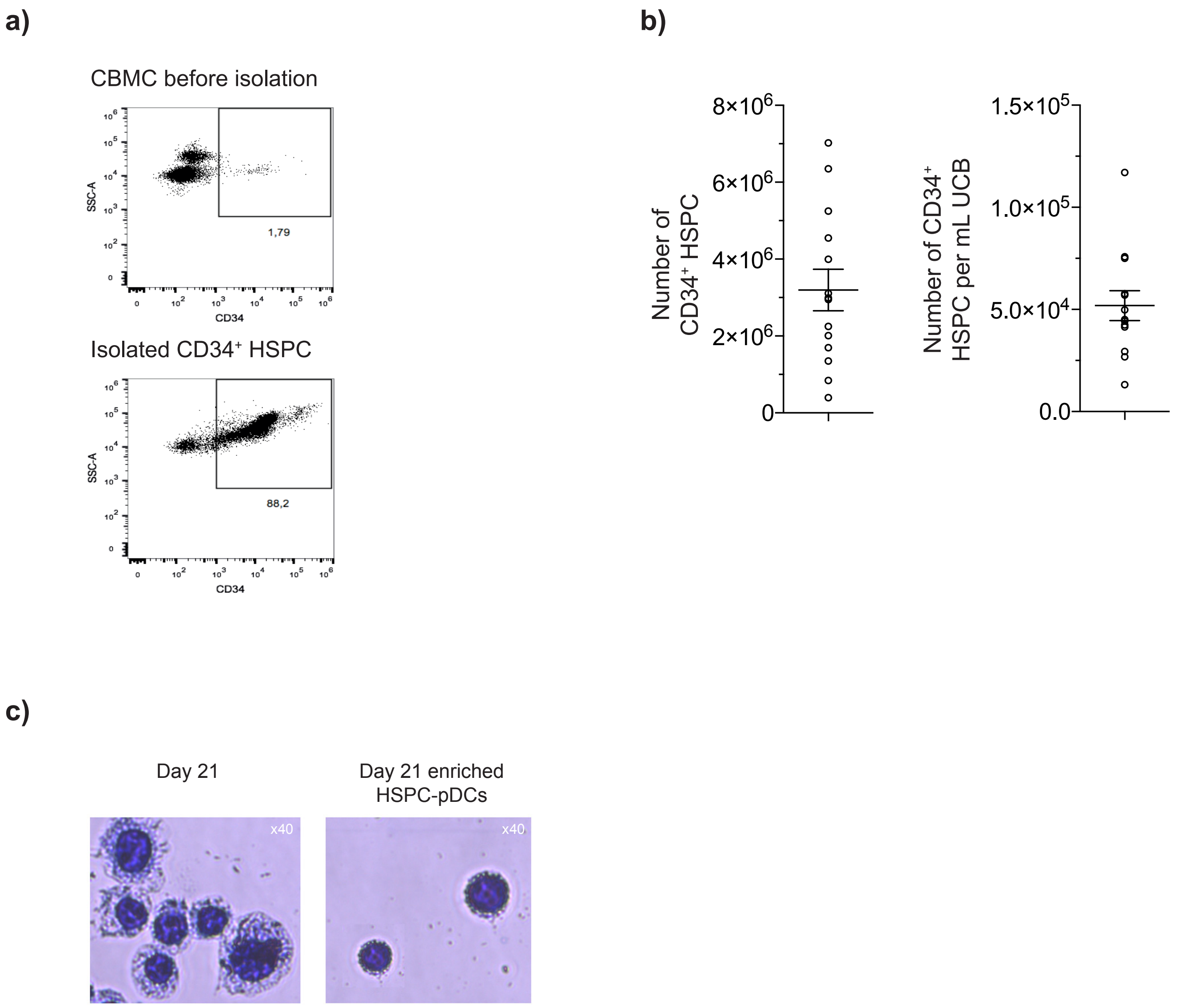

Generation of HSC-pDCs from HSPC. a) Flow cytometric analysis of CD34 surface expression on cord-blood derived mononuclear cells (CBMC) before and after isolaion of CD34⁺ cells. **b**) Yield of CD34⁺ HSC in total numbers (left) or per mL of umbilical cord blood (right). Data are ± SEM and each dot represent a single donor of 13 donors. **c**) May-Grünwald-Giemsa stain of cultured cells at 21 day before or after enrichment of HSPC-pDCs.

Supplementary Figure 2

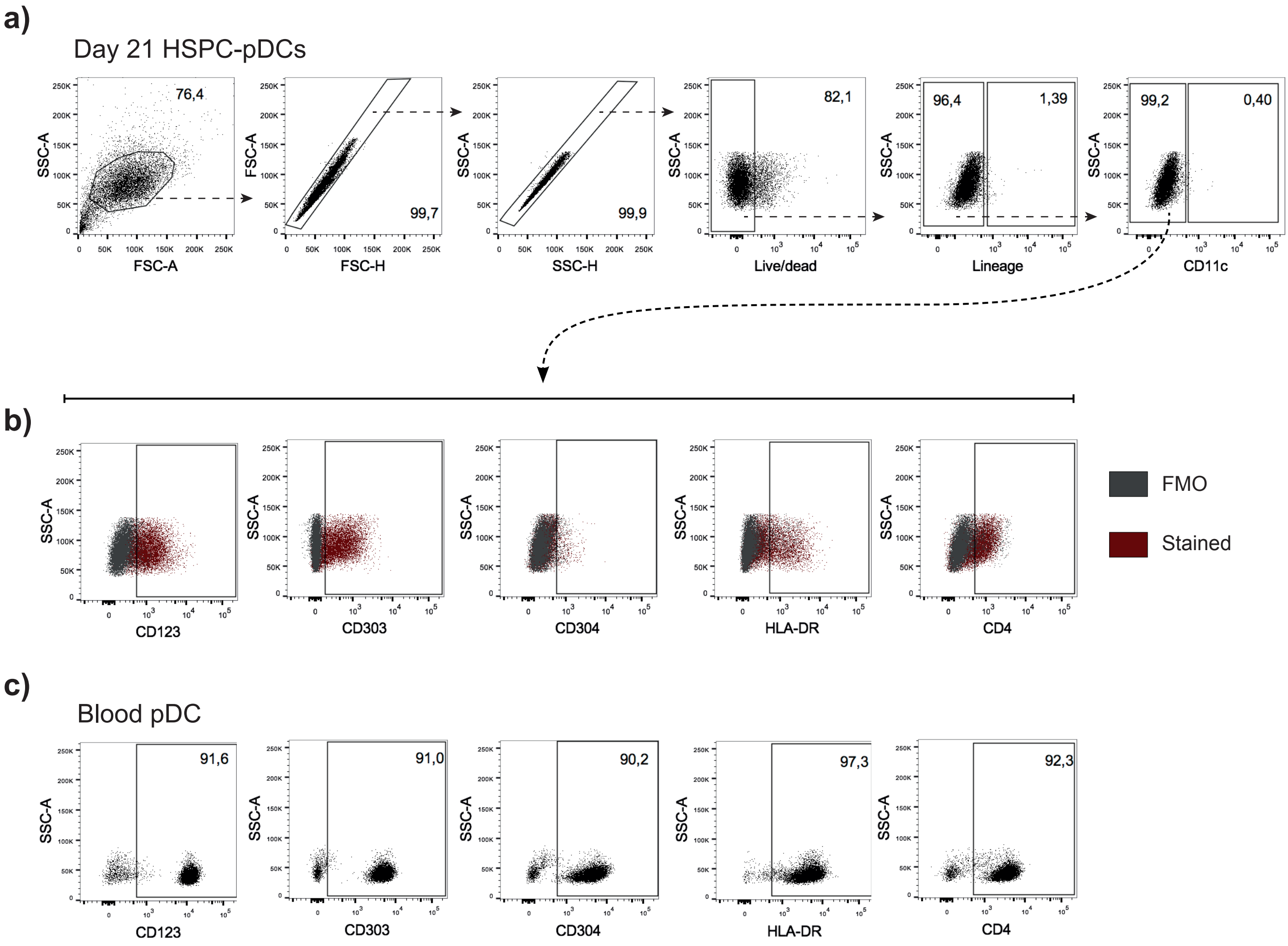

Gating strategy of HSPC-pDCs and blood pDCs. **a**) Gating strategy to discriminate HSPC-pDCs. **b**) Phenotypic analysis of CD123, CD303, CD304, HLA-DR and CD4. Cells were first gated based on FSC and SSC to exclude debris, and then doublets were excluded in regards to FSC-A and SSC-A. A viability stain, a lineage stain (lin) and a CD11c stain was next used to gate on viable/lin^{neg}/CD11c^{neg} cells for the indicated pDC-related markers. Fluorescence minus one (FMO) controls were utilized to set the gates for the positive/negative events. **c**) Phenotypic analysis of blood pDC with the same gates as determined in b).

Supplementary Figure 3

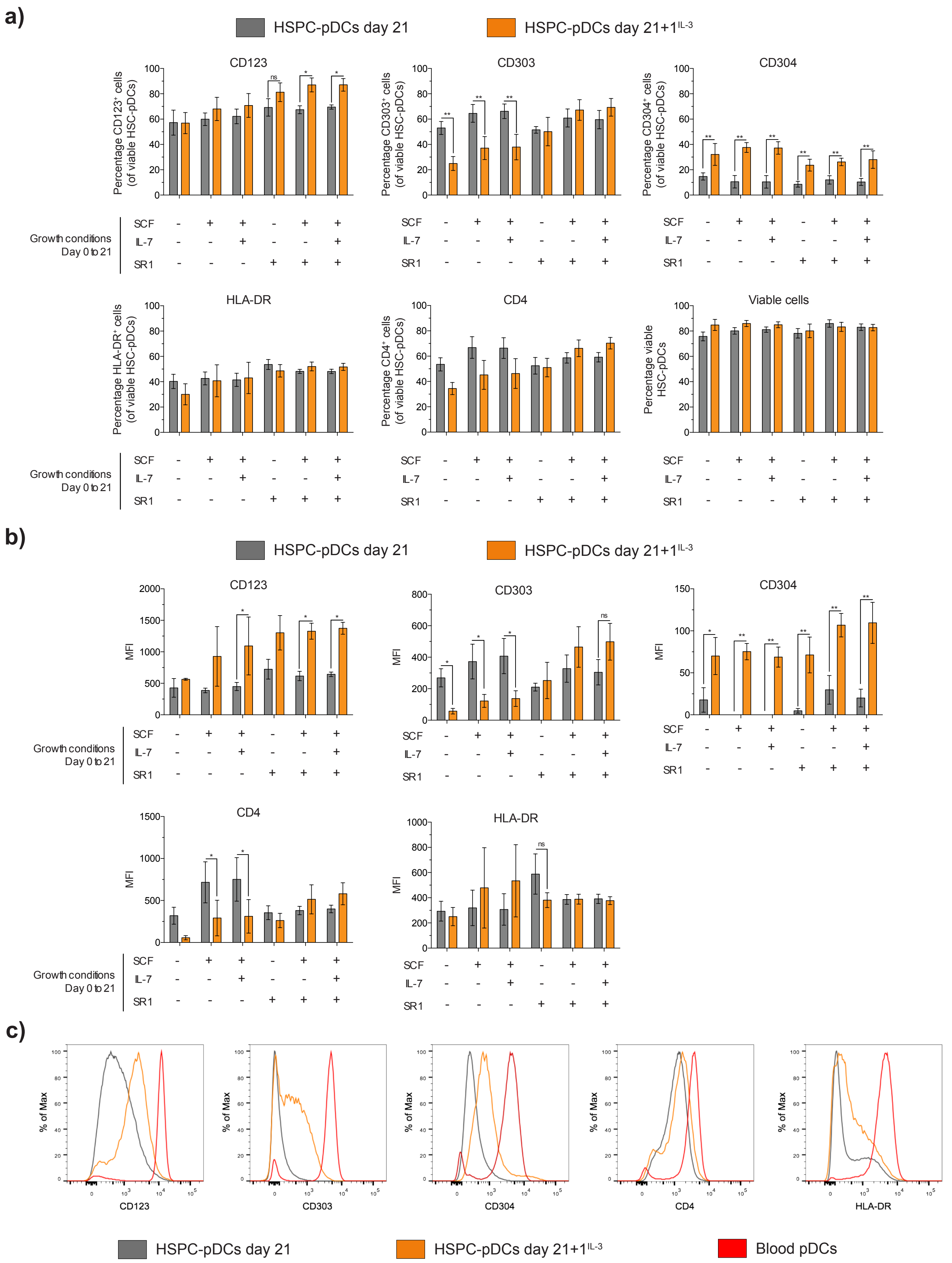

Removal of growth factors promote the up-regulation of pDC specific markers on HSPC-pDCs. Expression levels of CD123, CD303, CD304, HLA-DR or CD4 on HSPC-pDCs at day 21 or after one day of culture in medium depleted for growth factors (day 21+1^{IL-3}) for all tested growth conditions. Levels are either depicted as percentages of all viable cells (**a**) or as absolute MFI values (**b**). **c**) Representative histograms showing the expression of CD123, CD303, CD304, CD4 and HLA on HSPC-pDCs at day 21, at day 21+1^{IL-3} and blood pDCs. Data are ± SEM of four donors. Statistical analysis was performed using regular two-way ANOVA (a-b) followed by Bonferroni post hoc test.

Supplementary Figure 4

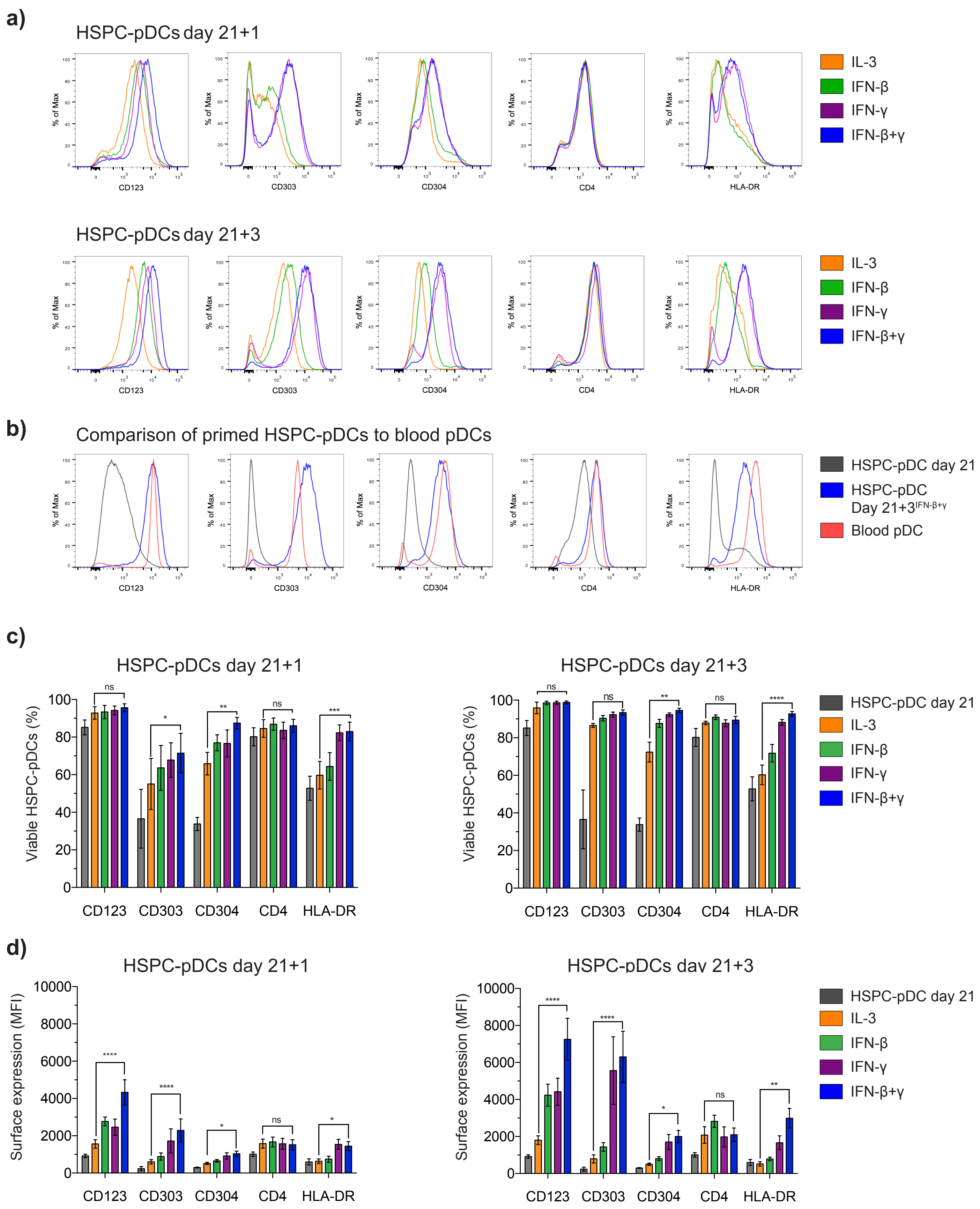

Priming of HSPC-pDCs up-regulates the surface expression of pDC-related markers. **a**) Phenotypic analysis of HSPC-pDCs at day 21+1 or day 21+3 under indicated priming conditions. **b**) Phenotypic comparison of HSPC-pDCs at day 21, IFN-β+γ primed HSPC-pDCs at day 21+3 and blood pDC. **c, d**) Phenotypic comparison of different priming condition on HSPC-pDCs, either depicting the percentage of positive cells (**c**) or absolute MFI values (**d**). Each bar shows the mean of five donors ± SEM. Statistical analysis was performed using regular two-way ANOVA (c-d) followed by Bonferroni post hoc test.

Supplementary Figure 5

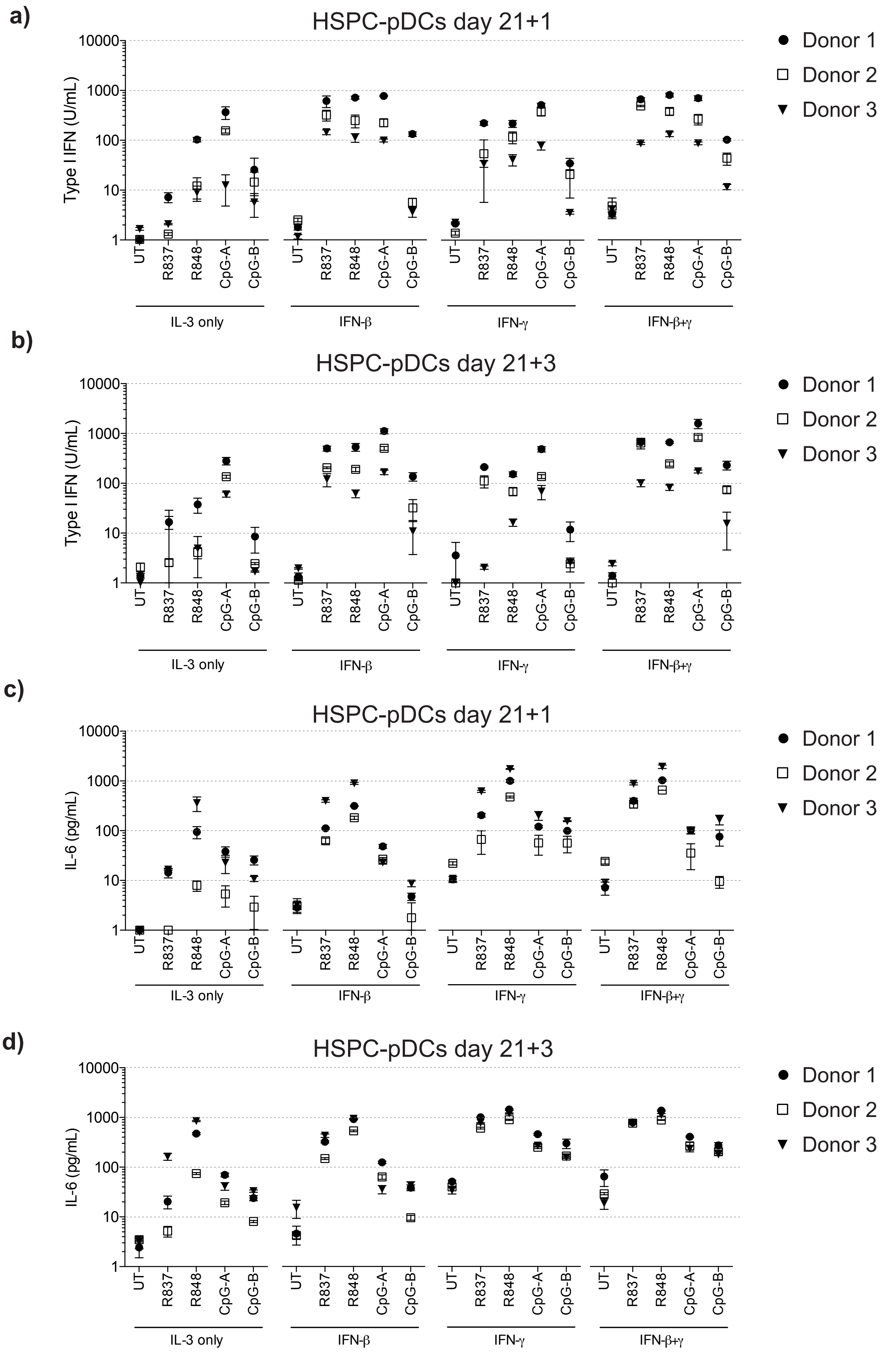

IFN priming increases the capacity of HSPC-pDCs to induce a type I IFN or pro-inflammatory IL-6 upon to TLR agonists. a-b) Levels of functional type I IFN in response to TLR7 and TLR9 agonists of IFN-primed HSPC-pDCs at day 21+1 (**a**) and day 21+3 (**b**). **c-d**) Levels of IL-6 produced in response to TLR7 and TLR9 agonists of primed HSPC-pDCs at day 21+1 (**c**) and day 21+3 (**d**). Each dot represents individual donors. Data are ± SEM of triplicates from three donors

Supplementary Figure 6

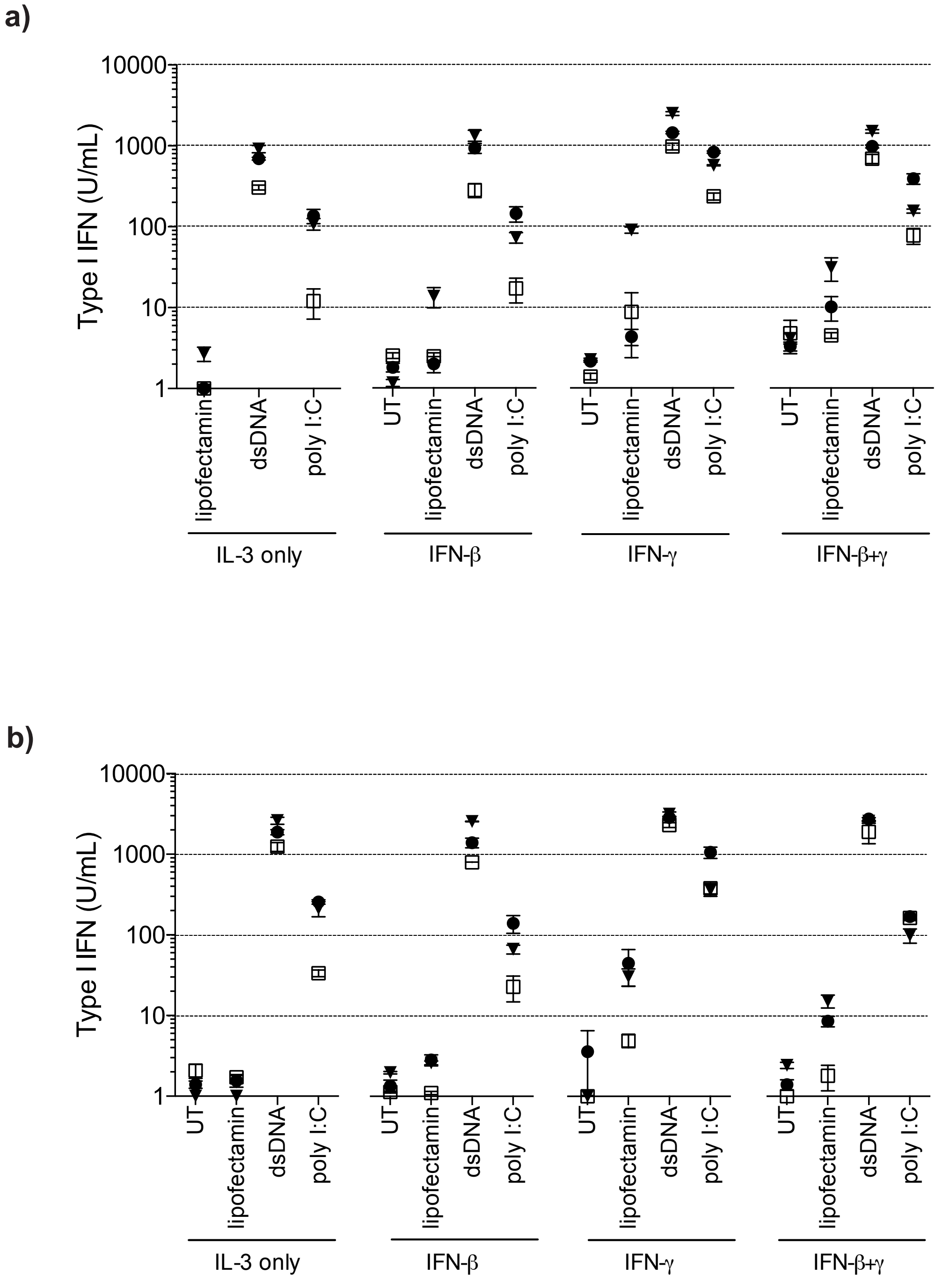

Priming of HSPC-pDCs increases the functional type I IFN response to cytosolic RNA/DNA agonists. a, b) Type I IFN produced in response to lipofectamin-formulated dsDNA and poly I:C of primed HSPC-pDCs at day 21+1 (**a**) and day 21+3 (**b**). Each dot represents individual donors. Data are ± SEM of triplicates from three donors.

Supplementary Figure 7

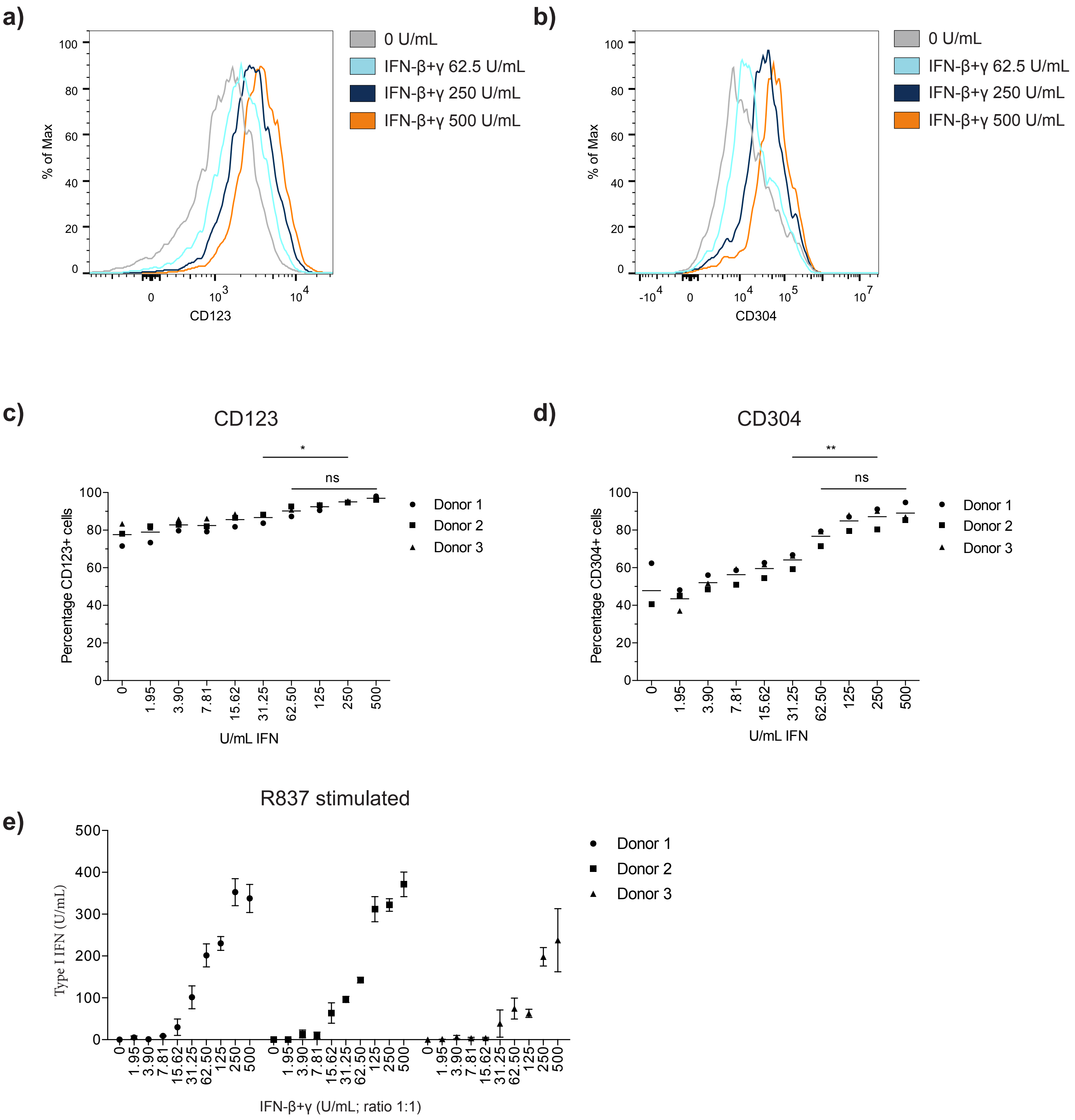

Titration of Interferon on HSPC-pDCs. HSPC-pDCs were primed with varying concentrations of IFN-β+γ ranging from 1.95 to 500 U/mL or left unprimed (0 U/mL). After three days cells were phenotypically analyzed for the expression of CD123 and CD304 using flow cytometry or stimulated with R837 for functional validation of the type I IFN response. **a, b**) Phenotypic analysis of CD123 (left) or CD304 (right) of unprimed or HSPC-pDCs primed with either 62.5, 250 or 500 U/mL of IFN-β+γ. **c, d**) Phenotypic analysis of HSPC-pDCs at day 21+3 under indicated priming conditions. Column diagrams show percentage of CD123 or CD304 (**c, d**). **e**) Type I IFN produced in response to the TLR7 agonist R837 of primed HSPC-pDCs at day 21+3 for each donor tested. Each dot represent individual donors. Data are ± SEM of singlets (**a-d**) or triplicates (**e**) of three donors. Statistical analysis was performed using regular two-way ANOVA (c-d) followed by Bonferroni post hoc test.

Supplementary Figure 8

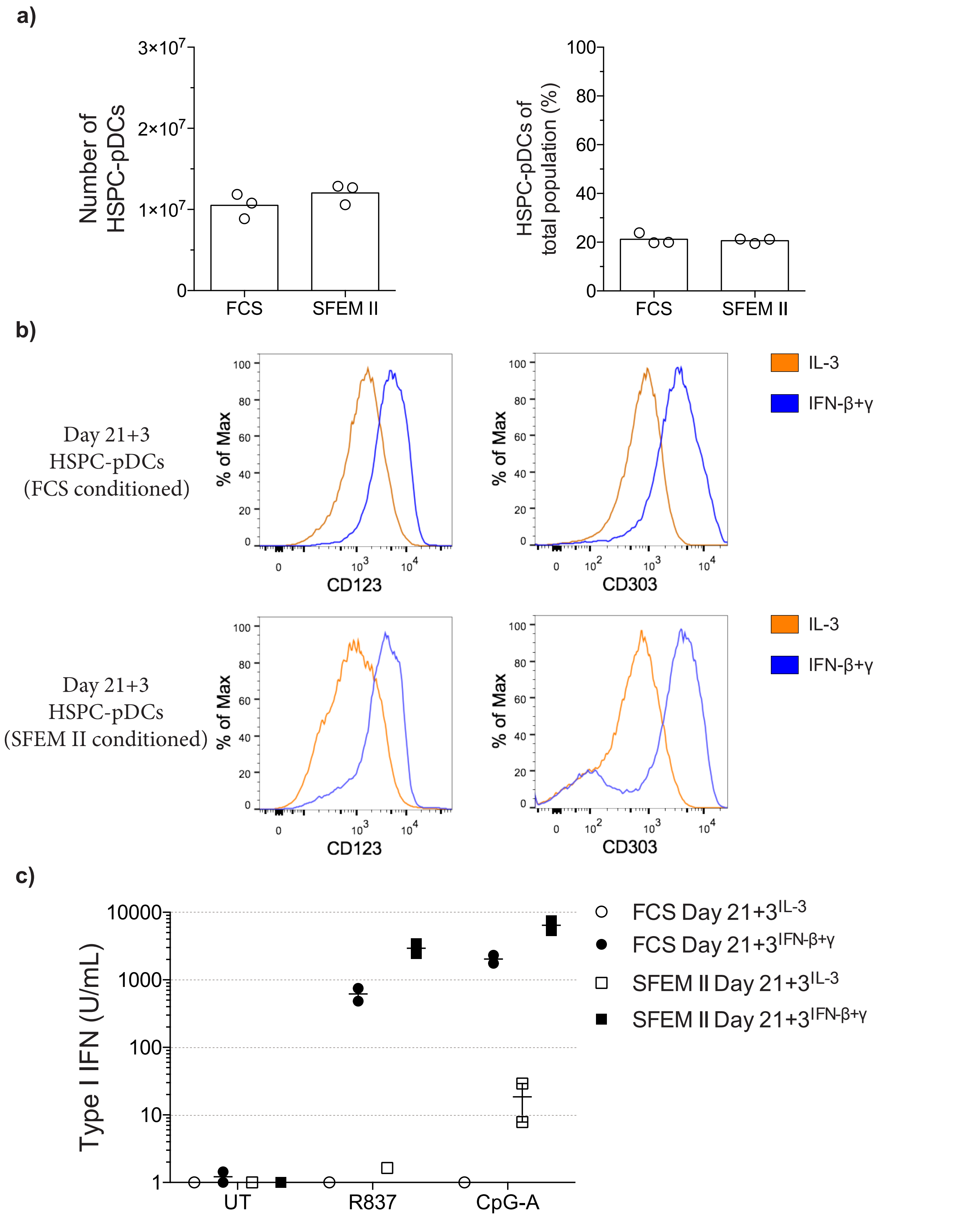

HSPC-pDCs can be generated using serum-free conditions. **a**) Numbers and percentage of HSPC-pDCs generated using serum-free conditions (SFEM II) or RPMI medium supplemented with FCS. **b**) Phenotypic comparison of primed and unprimed HSPC-pDCs (viable, lineage$^-$CD11c$^-$ cells) at day 21+3. Upper panel show HSPC-pDCs generated using RPMI medium supplemented with FCS and lower HSPC-pDCs generated using SFEM II. **c**) Type I IFN produced in response to TLR7 and TLR9 agonists of primed and unprimed HSPC-pDCs derived from HSPC cultured in SFEM II or RPMI medium supplemented with FCS. Data shown are from three donors (**a**), two donors (**b**) and two donors done in biological dublicates (**c**).

Supplementary Figure 9

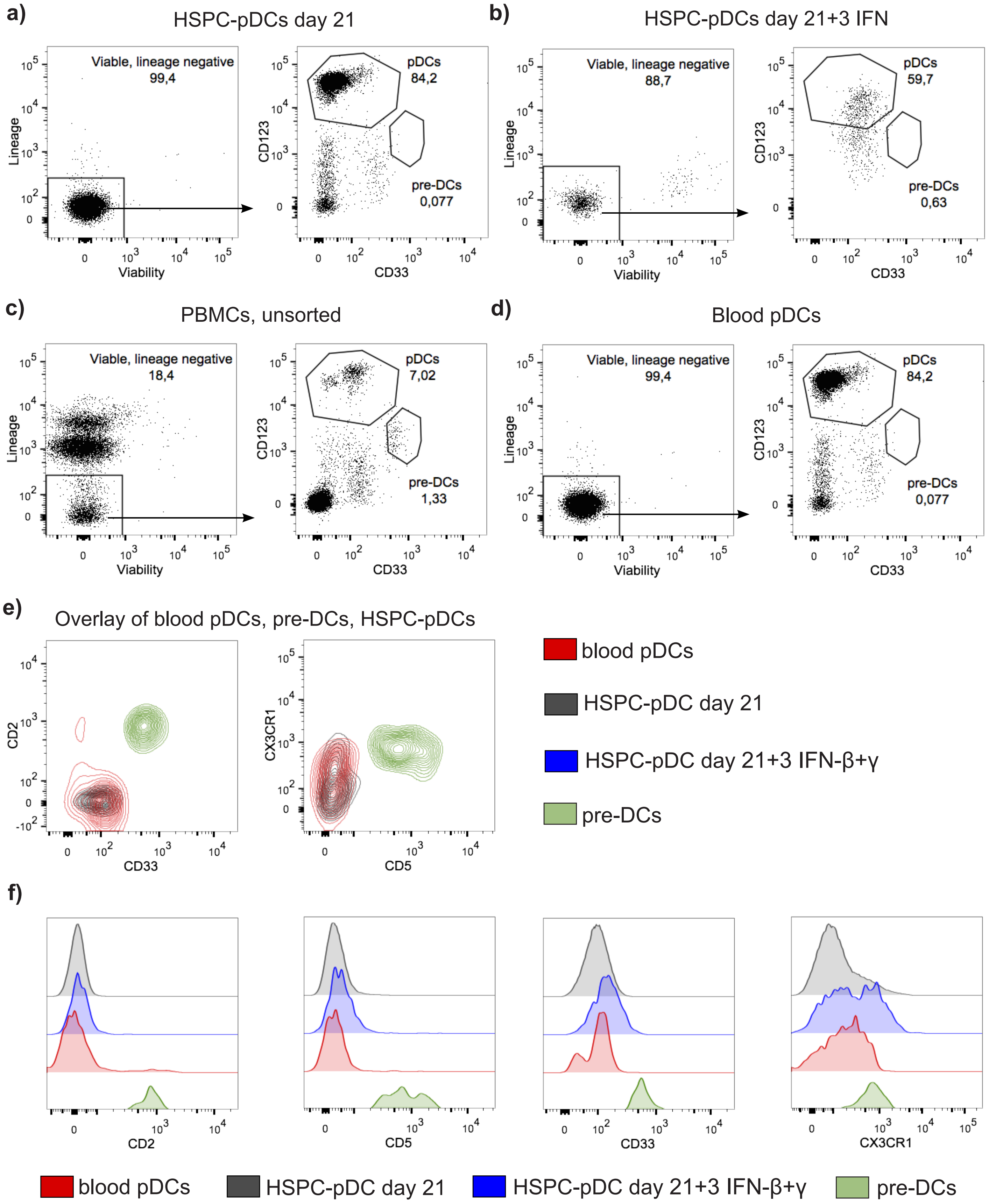

Pre-DCs are absent in the HSPC-pDCs population. a-d) Dot plot showing the expression of CD123 vs. CD33 of HSPC-pDCs at day 21 (**a**), IFN primed HSPC-pDC at day 21+3 (**b**), PBMCs before pDC enrichment (**c**) and enriched blood pDCs (**d**). **e**) Phenotypic comparison pre-DCs, blood pDCs, HSPC-pDCs at day 21 and IFN primed HSPC-pDC at day 21+3 using overlaid 2D-contour plots. **f**) Overlaid histograms showing the expression of CD2, CD5, CD33 and CX3CR1 for phenotypc comparison of pre-DCs, blood pDCs, HSPC-pDCs at day 21 and IFN primed HSPC-pDCs at day 21+3. Data are singlets of three donors.

Supplementary Figure 10

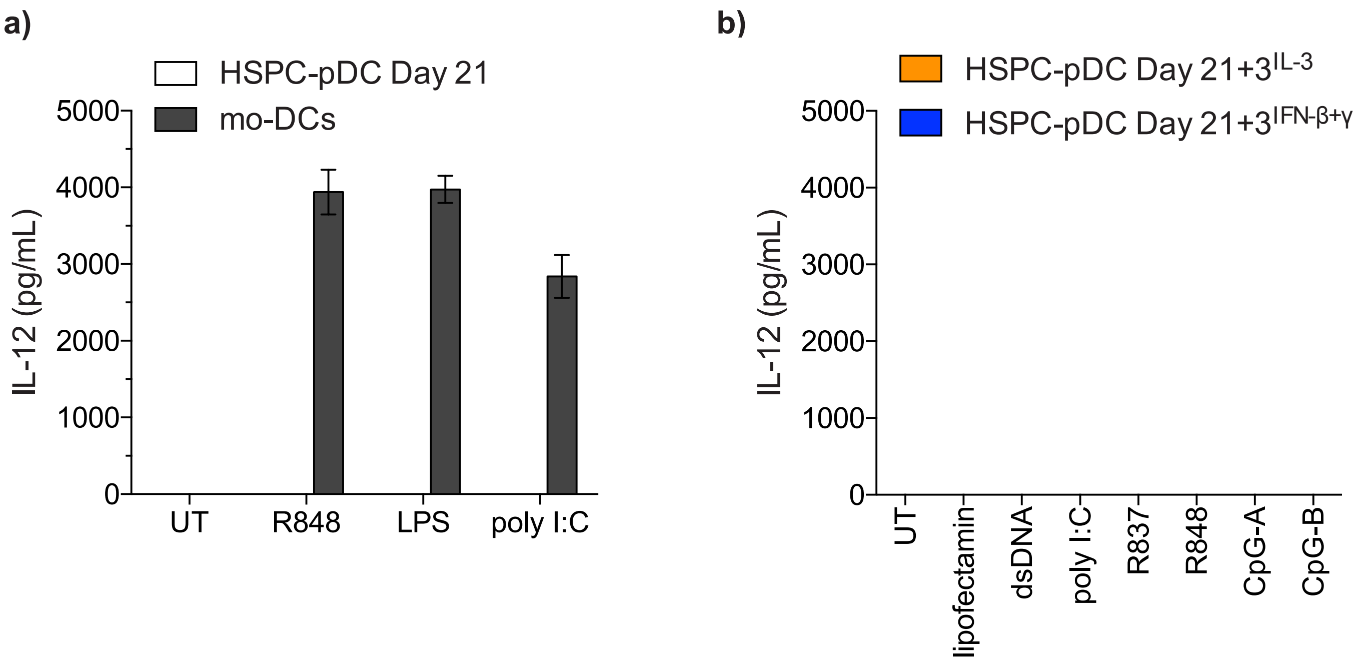

Capacity of HSPC-pDCs to produce IL-12 upon stimulation cDC activating agonists. **a**) Levels of IL-12 in response to the cDC activating stimuli R848, poly I:C and LPS in HSPC-pDCs at day 21 and mo-DCs. **b**) Levels of IL-12 in primed and unprimed HSPC-pDCs at day 21+3 in response to TLR7, TLR9 or cytosolic DNA/RNA agonists (supernatants are from the same donors used in Figure 3c-d and Supplementary Fig. 5-6). Data are ± SEM of dublicates from two donors (**a**) and three donors in triplicates (**b**).

Supplementary Figure 11

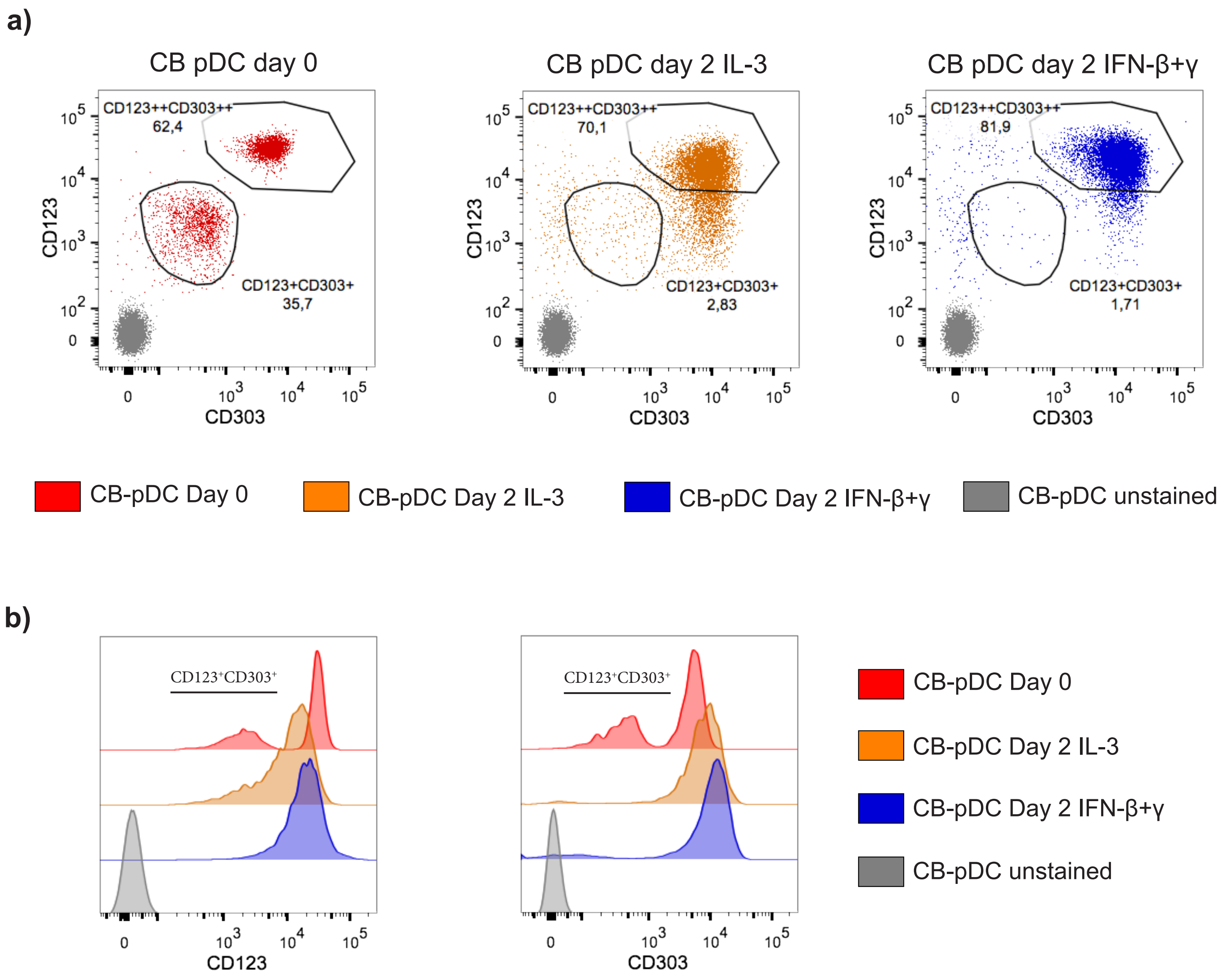

A population of cells within human CB elicit an intermediate expression of pDC markers. a) cord blood mononuclear cells were enriched for pDCs and cells were either phenotypically analyzed for expression of pDC markers, or primed for two days under indicated conditions before being phenotypically analyzed. Dot-plots show expression of CD123 versus CD303 on viable, lineage negative and CD11c negative cells. **b**) Off-set histograms showing expression levels of CD123 and CD303 for the identified population in (**a**) for the different conditions. An unstained sample for CB-pDC at day 0 has been included to separate the positive and negative populations. Data are representative from three donors

Supplementary Figure 12

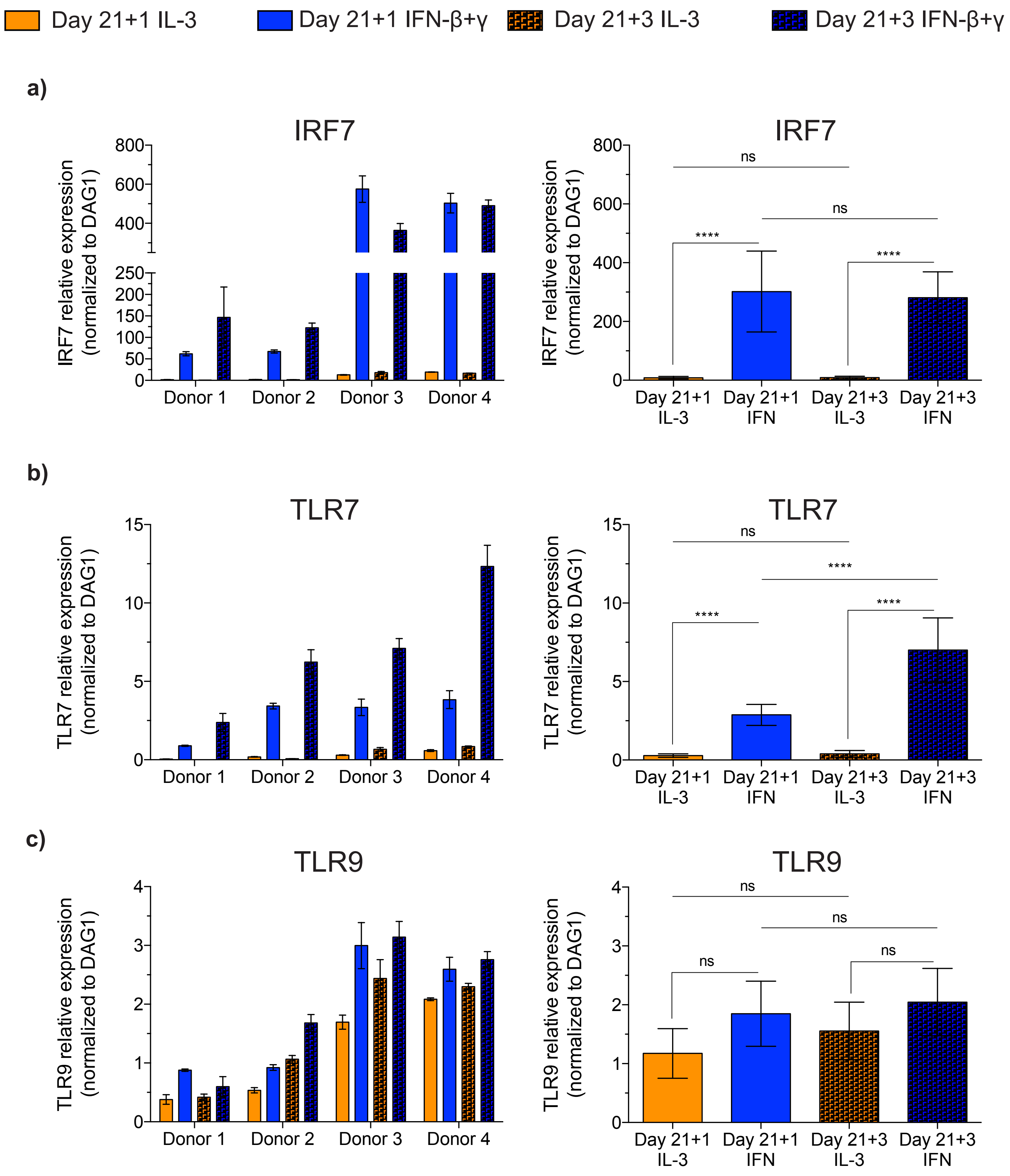

Expression levels of genes of primed and unprimed HSPC-pDCs. HSPC-pDCs were primed with IFN-β+γ or were left unprimed with IL-3 alone. After one day (day 21+1) or three days (day 21+3), cells were lysed and the gene expression level of IRF7 (**a**), TLR7 (**b**) or TLR9 (**c**) was assessed using qPCR. Data shown are ± SEM of biological triplicates from four donors, either shown as individual (right) or collective (left) donors. Statistical analysis was performed using regular two-way ANOVA followed by Bonferroni post hoc test.

Supplementary Figure 13

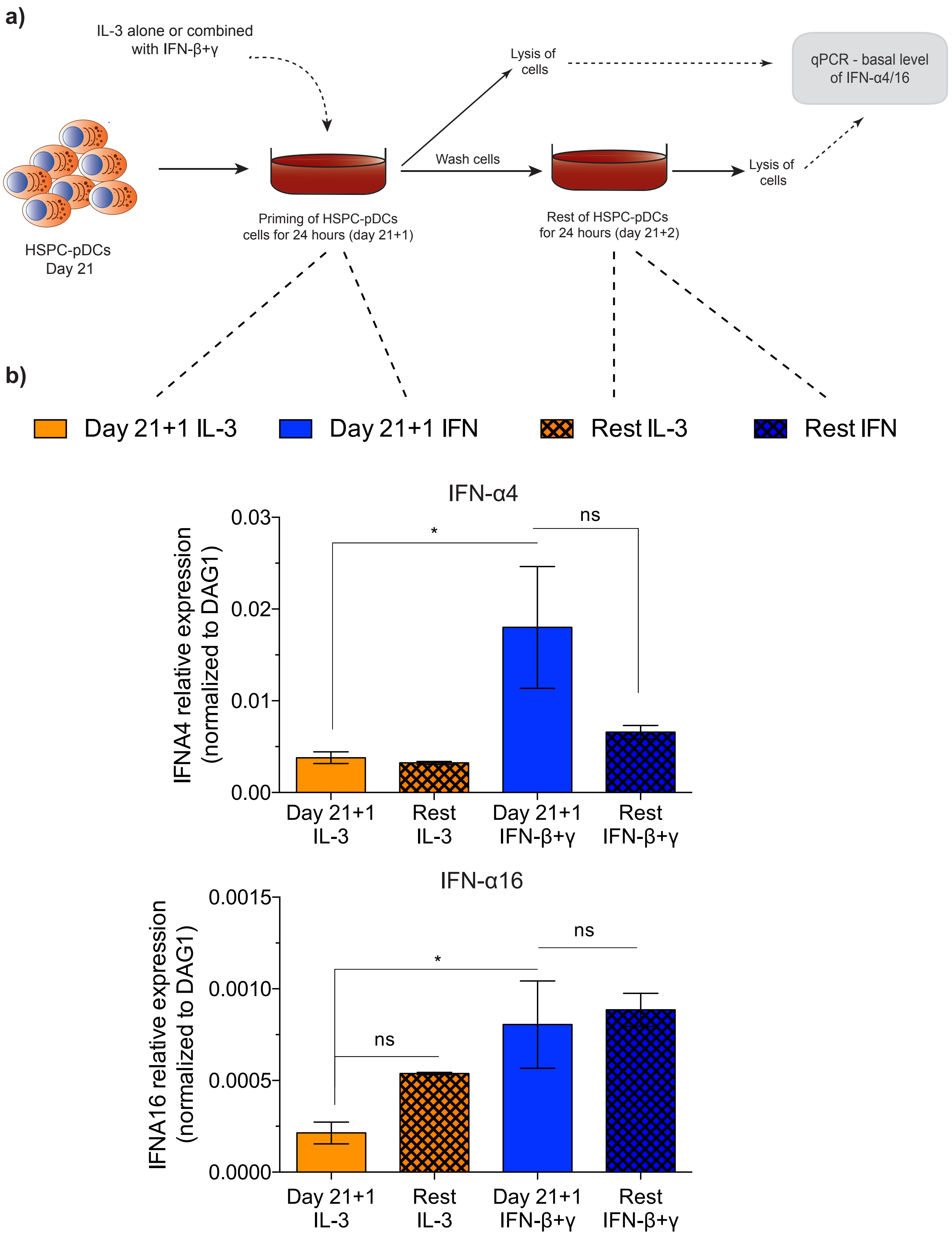

Expression levels of IFN-α2, IFN-α4 and IFN-α16 after resting phase of HSPC-pDCs. a) Graphical illustration of resting of HSPC-pDCs. Cells were primed or left unprimed. After 24 hours (Day21+1) cells were either lysed or washed and resuspend in medium with IL-3 alone and cultivated for an additional 24 hours (rest) before being lysed. Basal expression levels of selected IFN-α genes was subsequently evaluated using qPCR. **b**) Basal expression levels of IFN-α4 and IFN-α16. Data are ± SEM of biological triplicates from two donors. Statistical analysis was performed using regular two-way ANOVA (b) followed by Bonferroni post hoc test.

Supplementary Figure 14

a)

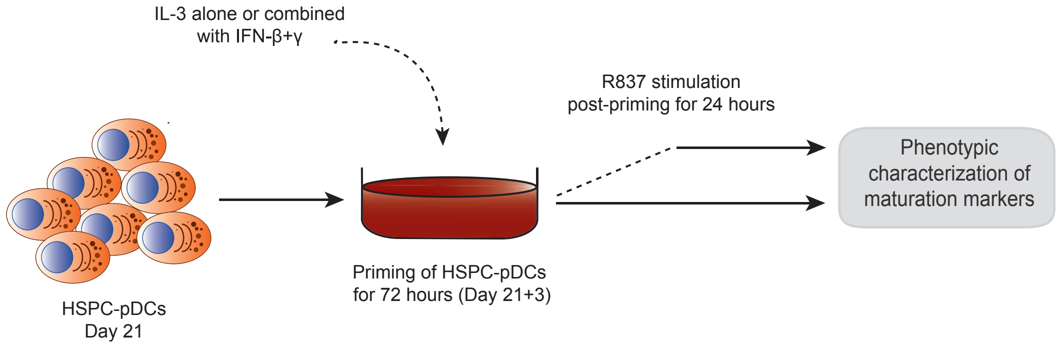

b)

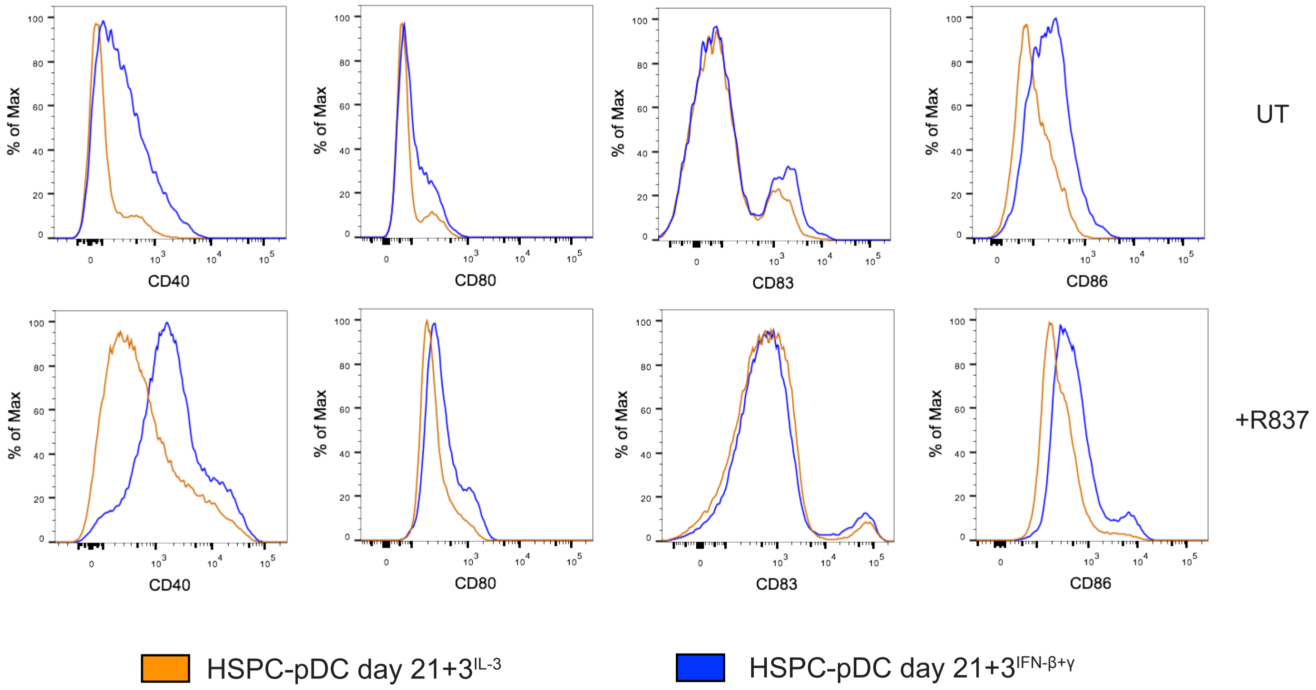

TLR7 stimulation of primed HSPC-pDCs up-regulates co-stimulatory markers. a) Graphical illustration of the maturation process of HSPC-pDCs. Cells were primed for three days (day 21+3) with IFN-β+γ or left unprimed (IL-3). Cells were then either phenotypically analyzed or stimulated for an additional 24 hours with the TLR7 agonist R837 before phenotypic characterization. **b**) Phenotypic analysis of CD40, CD80, CD83 and CD86 of unprimed or primed HSPC-pDCs stimulated with R837 (lower) or left untreated (UT) (upper panel).

Supplementary Figure 15

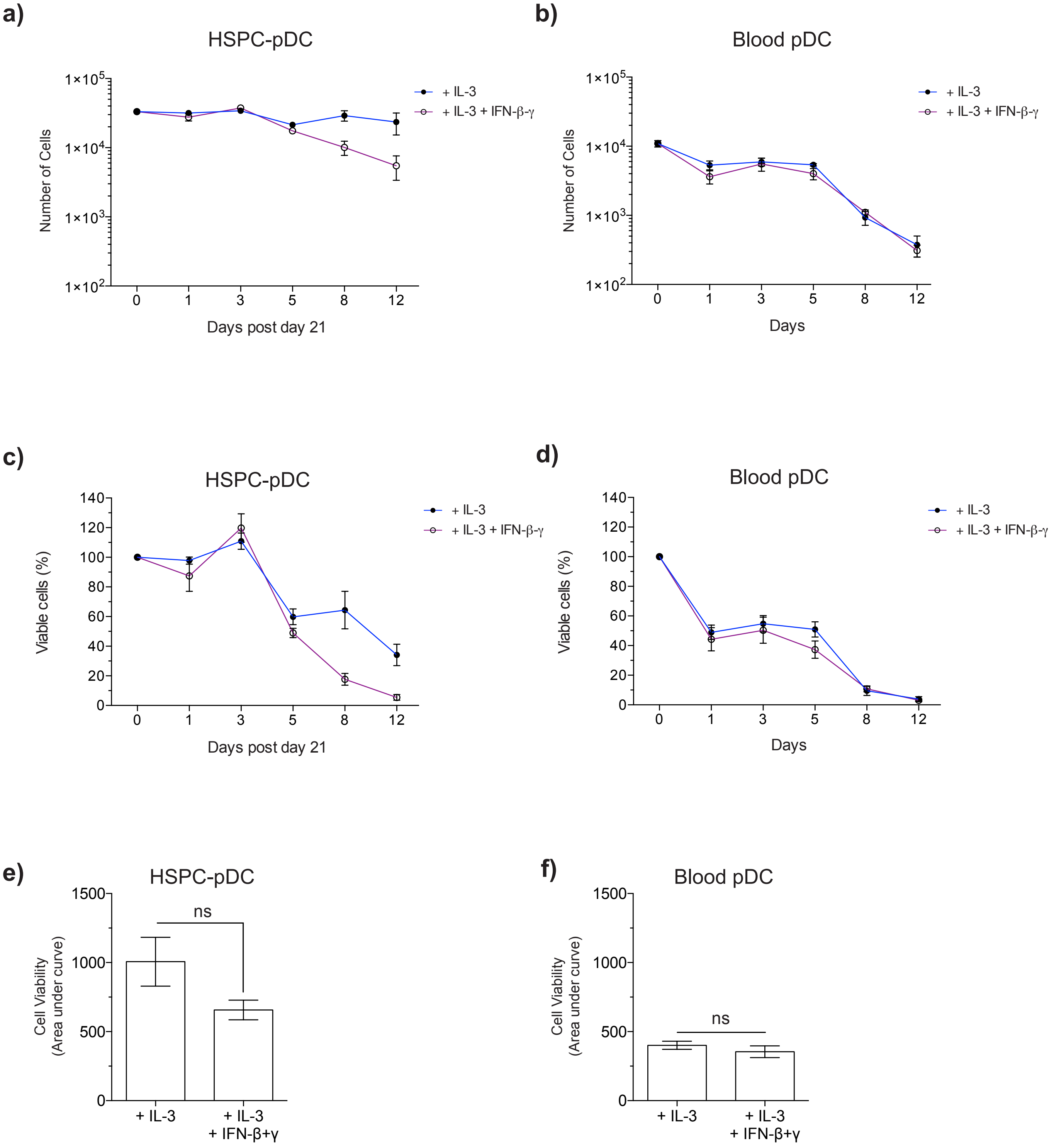

Viability of HSPC-pDCs and blood pDCs upon prolonged culture. a, b) HSPC-pDCs and blood pDCs were cultivated for 1, 3, 5, 8 and 12 days in medium supplemented with IL-3 or IL-3 + IFN-β+γ. At each time point number of viable cells were assessed using flow cytometry. **c, d**) Percentage of viable cells over prolonged culture calculated from panel (**a**) and (**b**). **e, f**) Area under curve calculated from (**c**) and (**d**). Data are ± SEM of biological duplicates (for blood pDCs) or triplicates (for HSPC-pDCs) from four donors at each time-point. Statistical analysis was performed using regular one-way ANOVA (e-f) followed by Bonferroni post hoc test.

Supplementary Figure 16

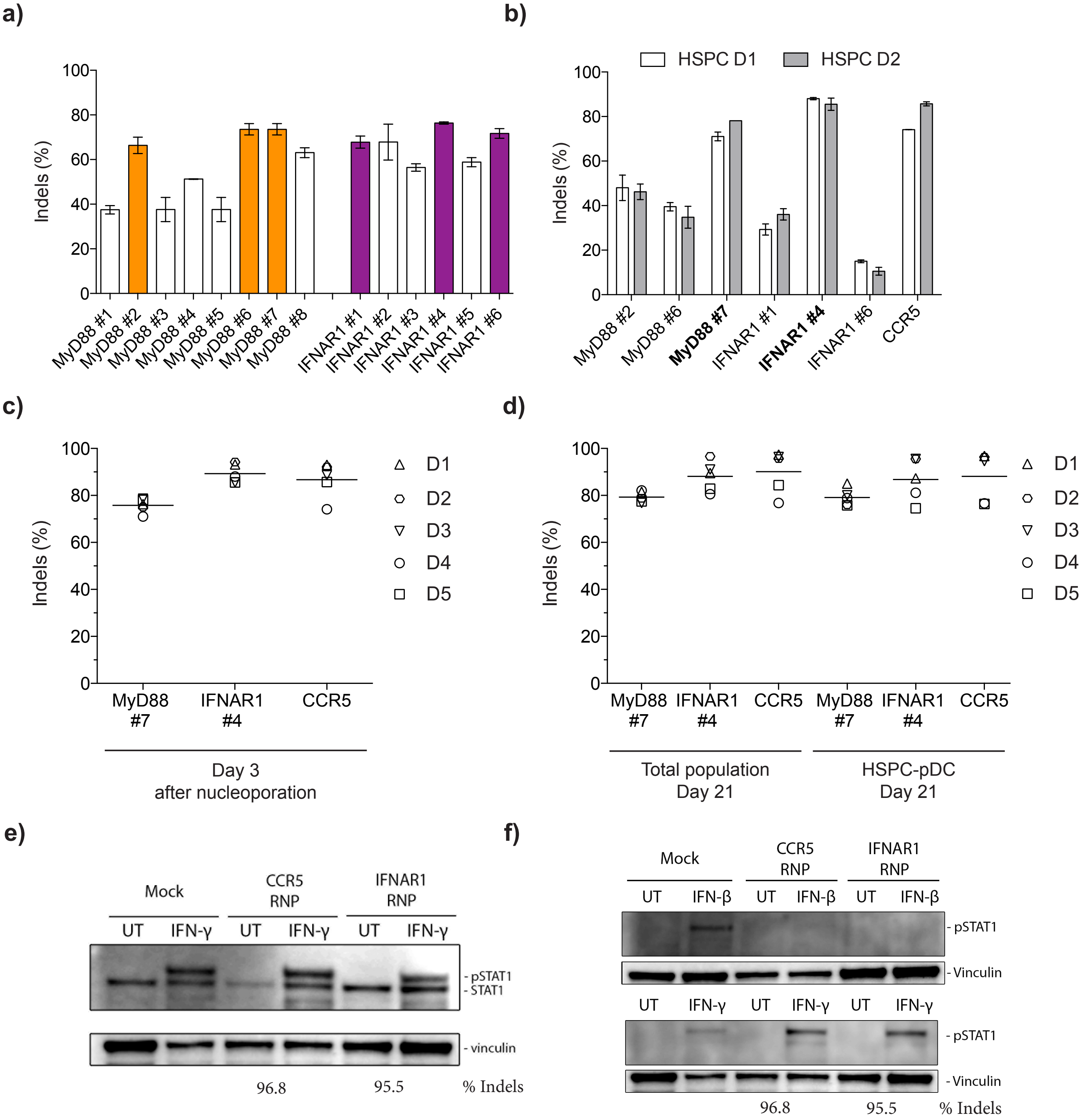

Genetic modifications are retained during cultivation and differentiation of HSPCs into HSPC-pDCs. a) sgRNAs were initially screened in K562 cells by electroporation of sgRNA-px330 plasmid. From this screening, 3 sgRNAs with the highest potency to induce Indels for MyD88 (orange; #2, #6 and #7) and IFNAR1 (purple; #1, #4 and #6) were selected for further validation. **b**) Selected sgRNAs were ordered as chemically modified sgRNAs and subsequently screened in CD34^{+} HSPC alongside sgRNAs directed at exon one of CCR5 (control). sgRNAs with high potency (MyD88 #7 and IFNAR1 #4) were selected for further investigation. **c**) Indel frequencies of HSPCs three days after electroporation with sgRNAs directed against MyD88, IFNAR1 and CCR5. **d**) Indel frequencies of the total population of cells or the enriched fraction of HSPC-pDCs at day 21 of culture for HSPCs electroporated with sgRNAs. Indel frequencies were measured by TIDE analysis using a mock transfected sample as reference control. **e**) Induction of pSTAT1 (indirectly measured by upper shifted band) upon stimulation with IFN-γ in HSPC-pDC targeted with sgRNAs directed against IFNAR1, CCR5 or mock (detection Ab: STAT1 [CST 9172]). **f**) Induction of pSTAT1 upon stimulation with IFN-β or IFN-γ in HSPC-pDC targeted with sgRNAs directed against IFNAR1, CCR5 or mock (detection Ab: pSTAT1 [CST 7649]). Indel frequencies at the respective target are listed below the Western Blot. Bars represent average Indel frequencies ± SEM of two experiments (**a**),two donors (**b**) done in biological duplicates or 5 donors in singlets (**c, d**).

Supplementary Figure 17

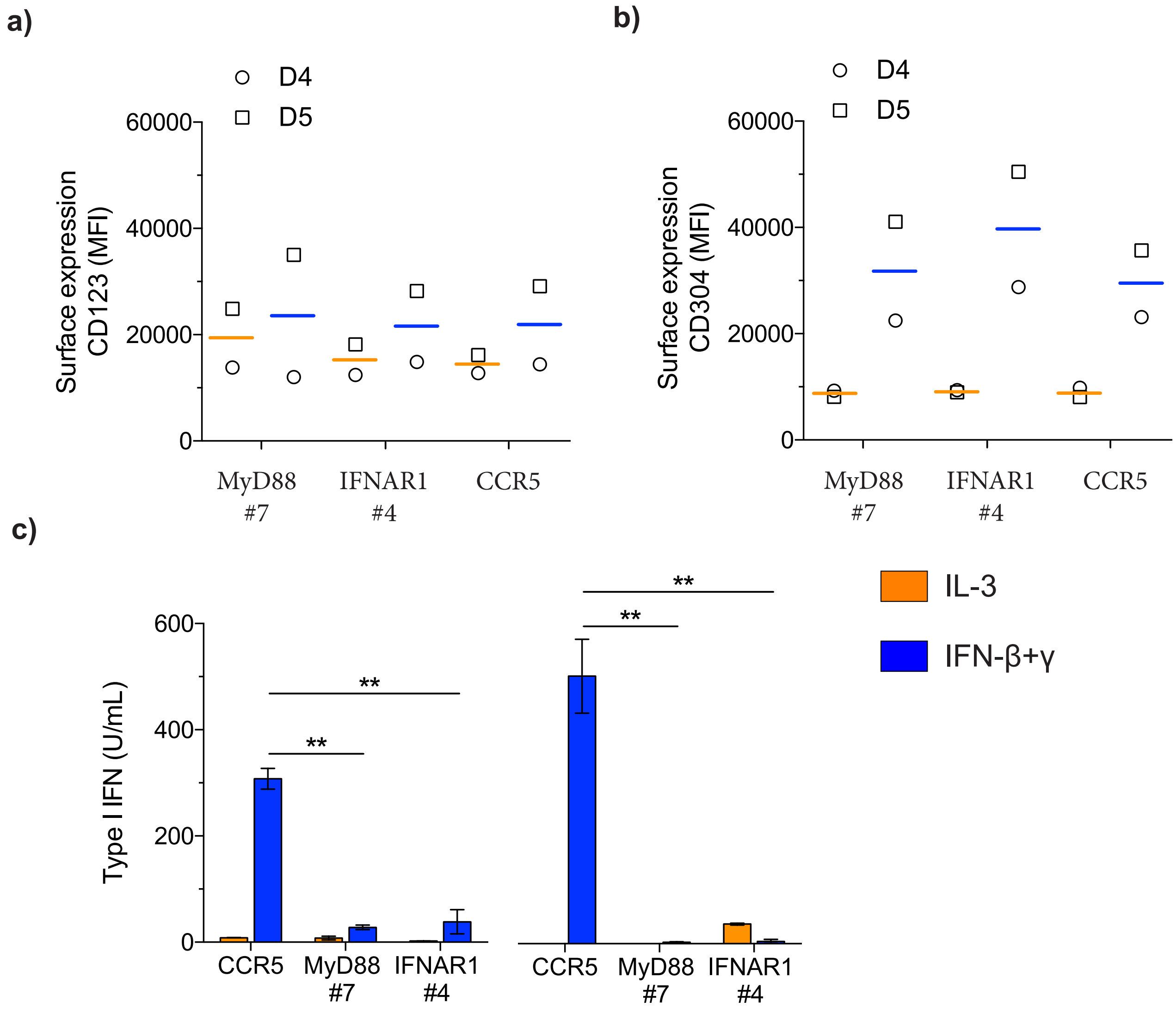

Phenotypic and functional analysis of knock-down of MyD88, IFNAR1 and CCR5 of two additional donors. **a, b**) Surface expression levels (MFI) of CD123 (**a**) and CD304 (**b**) in primed and unprimed HSPC-pDCs with gene editing at CCR5, MyD88 and IFNAR1. **c**) Functional levels of type I IFN after stimulation with R837 (TLR7) or CpG-A (TLR9) in unprimed and primed HSPC-pDCs gene-edited at CCR5, MyD88 or IFNAR1. Data are ± SEM of two donors. Statistical analysis was performed using regular two-way ANOVA (**c**) followed by Bonferroni post hoc test.

Supplementary Figure 18

Figure 5e
WB: MyD88 (Cell Signaling Technologies D80F5)

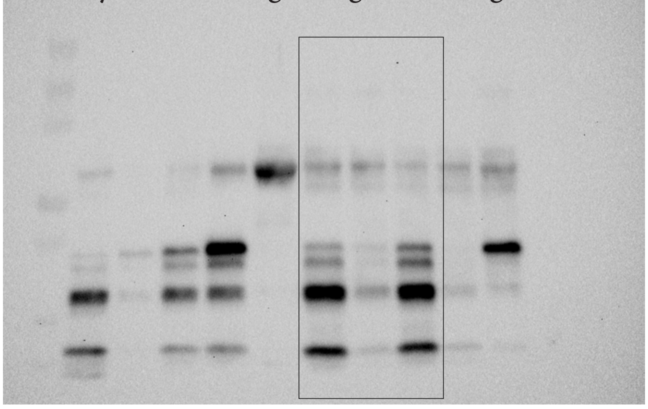

Figure 5e,
WB: Vinculin (Sigma-Aldrich hVIN-1)

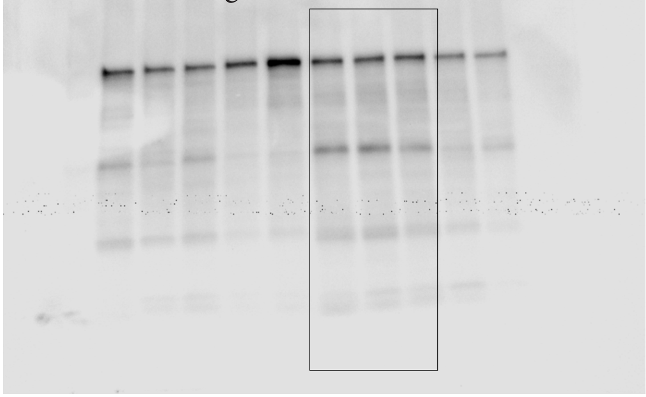

Figure 5e, Supplementary Figure 16e
WB: STAT1 (Cell Signaling Technologies #9172)

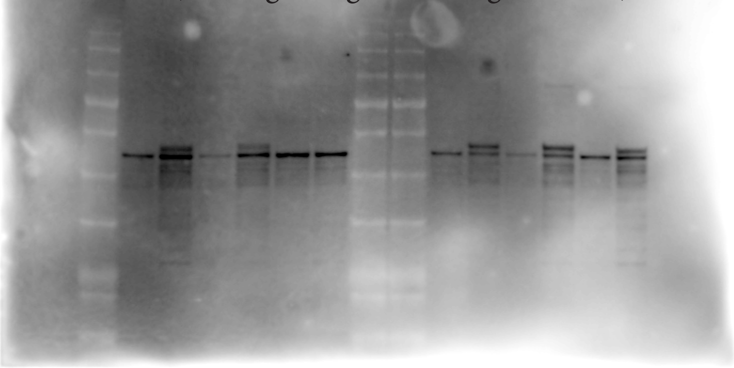

Figure 5e, Supplementary Figure 16e-f
WB: Vinculin (Sigma-Aldrich hVIN-1)

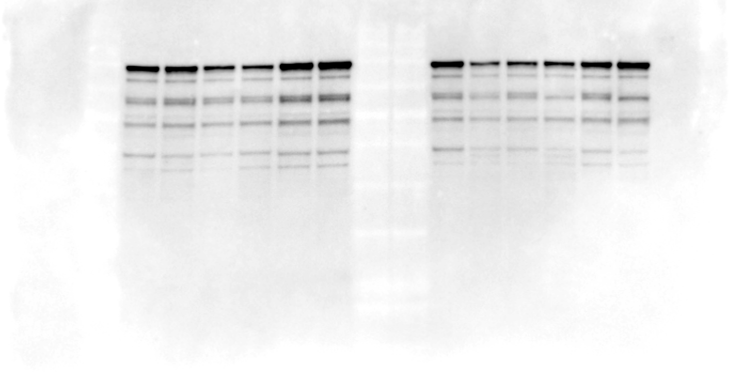

Supplementary Figure 18

Supplementary Figure 16f
WB: pSTAT1 (Cell Signaling Technologies #7649)

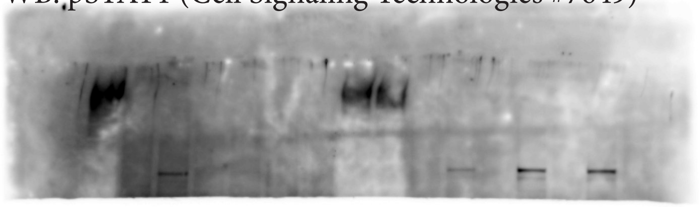

Supplementary Table 1. Agonists for functional evaluation of HSPC-pDCs

Agonist	Manufacturer	Cat.#	Targeted PRR	Concentration
R837 - imiquimod	InvivoGen	tlrI-imq	TLR7	2,5 µg/mL
R848 - resiquimod	InvivoGen	tlrI-r848	TLR7, TLR8	0,5 µg/mL
CpG ODN 2216 (Class-A)	InvivoGen	tlrI-2216	TLR9	12 µg/mL
CpG ODN 2006 (Class-B)	InvivoGen	tlrI-bw006	TLR9	12 µg/mL
poly I:C (LMW)	InvivoGen	tlrl-picw	RIG-I	0.5 µg/mL
dsDNA (HSV-60 mer)	InvivoGen	tlrl-hsv60n	Cytosolic DNA sensor	2 µg/mL
LPS-B5 Ultrapure	InvivoGen	tlrl-pb5lps	TLR4	100 ng/mL
CMV ProMix	ProImmune	PX-CMV	APC function	1 µg/mL

Supplementary Table 2. Antibodies used for phenotypic evaluation of HSPC-pDCs

Antibodies/viability dye	Fluorochrome	Clone	Manufacturer	Cat. #	Dilution*	Panel
Linage cocktail 1 (CD3, CD14, CD16, CD19, CD20, CD56)	FITC	CD3:SK7, CD14:MϕP9, CD16:3G8, CD19:SJ25C1, C20:L27, CD56:NCAM16.2	BD	340546	0.3x	1,4
CD11c	APC	B-ly6	BD	559877	0.5x	1
CD123	PE	6H6	eBioscience	12-1239-42	0.03x	1, 2,4
CD303a	PE-Cy7	201A	eBioscience	25-9818-42	1x	1
CD304	BV421	12C2	Biolegend	354514	0.03x	1, 2
HLA-DR	BV650	G46-6	BD	564231	0.3x	1
CD4	PerCP-Cy5.5	RPA-T4	BD	560650	0.3x	1
Viability (7-AAD)	7-AAD		BD	559925	0.5x	1
Zombie Aqua Fixable Viability dye	BV510		Biolegend	423101	1x	2
CD34	PE	4H11	eBioscience	12-0349-42	0.5x	
CD40	PE	5C3	BD	555589	1x	3
CD80	FITC	2D10	Biolegend	305205	1x	3
CD83	PE-Cy7	HB15e	BD	561132	1x	3
CD86	BV605	2331 (FUN-1)	BD	562999	1x	3
LIVE/DEAD Fixable Near-IR Dead cell stai	APC-H7		ThermoFisher Scientific	L34975	1x	4
CX3CR1	PE-Cy7	2A9-1	Biolegend	341611	1x	4
CD2	BV605	RPA-2.10	Biolegend	300223	1x	4
CD33	BV510	WM53	Biolegend	303421	1x	4
CD5	AF-700	UCHT2	Biolegend	300631	1x	4

*Dilution of which the antibody used in, in comparison to the concentration stated by manufacturer (estimated from antibody titration)

Supplementary Table 3. Overview of sgRNA

Name	Sequence	Name	Sequence
MyD88 #1	5'-GTTCTTGAACGTGCGGACAC-3'	IFNAR1 #1	5'-GACCCTAGTGCTCGTCGCCG-3'
MyD88 #2	5'-GCTGCTCTCAACATGCGAGTG-3'	IFNAR1 #2	5'-GGGCGCGACGACCCTAGTGC-3'
MyD88 #3	5'-ACTGGACCGCGCTGGCGGAGC-3'	IFNAR1 #3	5'-GCTCGTCGCCGTGGCGCCAT-3'
MyD88 #4	5'-GCTTGAACGTGCGGACACAGG-3'	IFNAR1 #4	5'-TAGTGCTCGTCGCCGTGGCGC-3'
MyD88 #5	5'-CGCTGAGGCTCCAGGACCGC-3'	IFNAR1 #5	5'-AGTGCTCGTCGCCGTGGCGC-3'
MyD88 #6	5'-CCTGTCTCTGTTCTTGAACGC-3'	IFNAR1 #6	5'-GTGCTCGTCGCCGTGGCGCCA-3'
MyD88 #7	5'-CTGGCTGCTCTCAACATGCGC-3'		
MyD88 #8	5'-GTGTCTCTGTTCTTGAACGTG-3'	CCR5 (control)	5'-GCAGCATAGTGAGCCCAGAA-3'

Supplementary Table 4. Primers for amplification of amplicons from genomic DNA (TIDE analysis)

Primer	Sequence	Primer	Sequence
MyD88 fwd	5'-CTCCGTGGAAGAACTGTGGC-3'	IFNAR1 fwd	5'- GGAGTCGTCCTGGAATGC-3'
MyD88 rev	5'- GGCGGCTGTATCCAACGC-3'	IFNAR1 rev	5'-ACCTCGAGAACTGACAATTATGC-3'
		CCR5 fwd	5'- GCACAGGGTGGAACAAGATGG-3'
		CCR5 rev	5'- CACCACCCCAAAGGTGACCGT-3'
